# Maternal Cannabis Use during Lactation and Potential Effects on Human Milk Composition and Production: A Narrative Review

**DOI:** 10.1016/j.advnut.2024.100196

**Published:** 2024-03-01

**Authors:** Irma Castro-Navarro, Mark A McGuire, Janet E Williams, Elizabeth A Holdsworth, Courtney L Meehan, Michelle K McGuire

**Affiliations:** 1 Margaret Ritchie School of Family and Consumer Sciences, University of Idaho, Moscow, ID, United States; 2 Department of Animal, Veterinary, and Food Sciences, University of Idaho, Moscow, ID, United States; 3 Department of Anthropology, the Ohio State University, Columbus, OH, United States; 4 Department of Anthropology, Washington State University, Pullman, WA, United States

**Keywords:** cannabis, breastfeeding, breastmilk, milk composition, cannabinoids, cannabinoid receptors, human milk, lactation, peroxisome proliferator-activated receptors, prolactin

## Abstract

Cannabis use has increased sharply in the last 20 y among adults, including reproductive-aged women. Its recent widespread legalization is associated with a decrease in risk perception of cannabis use during breastfeeding. However, the effect of cannabis use (if any) on milk production and milk composition is not known. This narrative review summarizes current knowledge related to maternal cannabis use during breastfeeding and provides an overview of possible pathways whereby cannabis might affect milk composition and production. Several studies have demonstrated that cannabinoids and their metabolites are detectable in human milk produced by mothers who use cannabis. Due to their physicochemical properties, cannabinoids are stored in adipose tissue, can easily reach the mammary gland, and can be secreted in milk. Moreover, cannabinoid receptors are present in adipocytes and mammary epithelial cells. The activation of these receptors directly modulates fatty acid metabolism, potentially causing changes in milk fatty acid profiles. Additionally, the endocannabinoid system is intimately connected to the endocrine system. As such, it is probable that interactions of exogenous cannabinoids with the endocannabinoid system might modify release of critical hormones (e.g., prolactin and dopamine) that regulate milk production and secretion. Nonetheless, few studies have investigated effects of cannabis use (including on milk production and composition) in lactating women. Additional research utilizing robust methodologies are needed to elucidate whether and how cannabis use affects human milk production and composition.

**Statement of Significance**

To our knowledge, no review has focused on the potential effects of maternal cannabis use on human milk composition and production. The evidence provided here supports the possibility that, through several well documented pathways, cannabinoids might alter lipid metabolism in the mammary gland, as well as milk production and secretion. Considering the recent increase in cannabis use among reproductive-aged women, this narrative review highlights the need for well-designed, focused studies to address this question.

## Methods

The research to write this narrative review was initially performed using PubMed and Google Scholar databases. The preliminary search included the terms “cannabis,” “marijuana,” “lactation,” “human milk,” and “breastfeeding.” Additional searches were conducted by looking for specific concepts addressed in each section. Articles cited in the papers obtained during the searches were also included. No restriction on the time of publication was established.

## Overall Trends in Cannabis Use

Cannabis (*Cannabis sativa*, *Cannabis indica*, and *Cannabis ruderalis*; often referred to collectively as marijuana) is one of the world’s most commonly used drugs, with >200 million people using it in 2020 [1]. Cannabis use is increasingly legalized in many regions around the globe, including numerous states in United States. California became the first state in the United States to allow medical use of cannabis in 1996. As of 2023, 21 states, 3 territories, and the District of Columbia have legalized recreational cannabis use, and an additional 16 states have regulated cannabis for medical use [2]. The use of this plant and its derivatives among adults has spread in conjunction with its legalization. In 2002, the Substance Abuse and Mental Health Services Administration reported that 6.2% of the population in United States used cannabis in the previous year [3]. Cannabis use in the United States has nearly tripled since that time and was reported to be 18.7% in 2021 with the greatest prevalence (35.4%) among young adults aged 18– 25 y [3].

Not only has the prevalence of cannabis use increased, but variation in cannabis products offered and the potency of those products is greater than ever. Historically, smoking dried cannabis flowers containing Δ-9-tetrahydrocannabinol (THC), the major psychoactive cannabinoid, was the most common method and product used. However, a notable variety of cannabis products, developed from processed raw cannabis, are now available (Table 1) [[4], [5], [6], [7], [8], [9], [10]], providing a wide diversity of modes of use [11]. Data from Washington State’s cannabis traceability system named Cannabis Central Reporting System (https://lcb.wa.gov/ccrs) shows that, although cannabis flower was still the most purchased product between 2014 and 2016 (accounting for two-thirds of expenditures), cannabis extracts sales increased 145% in that period [6].

**TABLE 1:** Summary of cannabis products, routes, and modes of use; average Δ-9-tetrahydrocannabinol concentrations; and average amount of Δ-9-tetrahydrocannabinol (mg) in a unit or package

Cannabis product^1^	Route of administration	Mode of use	THC concentration (THC % per weight of product) or total THC in product (mg/unit or mg/package)
Flowers (i.e., bud, dried herb)	Inhaled (smoked)	Cigarettes, joints, blunts, pipes, bongs	22% [4]; 14%–18% [5]; 21% [6]; 20% [7]
Hash, resin, kief	Inhaled (smoked)	Cigarettes, joints, blunts, pipes, bongs	44%, 71%, and 34%, respectively [4]; hash/kief 41% [7]
Liquid concentrates (oil, tinctures), dried herb	Inhaled (vaped)	Cartridges, vape pens	59%–74% [4]; 69% [6]; 68% [7]
Solid or semisolid concentrates (shatter, wax)	Inhaled (flash vaporized)	Dabs, slang	75% [4]; 73% [7]
Concentrates infused	Oral	Edibles (candy, baked goods, sublingual sprays, drinks, capsules)	2–8 mg/unit [4]; 0.01–99.1 mg/unit [8]; capsules 5–90 mg/unit; baked goods 8.4–50.6 mg/unit [9]; median through products = 10–25 mg/unit [10]
Concentrates infused	Transdermal	Lotions, balms, gels, patch	69 mg/unit [4]; 5 mg/unit [10]
Concentrates infused	Rectal or vaginal	Suppositories	240 mg/package [10]

1 Most popular cannabis products used by route/mode of administration; cannabis products can be adapted for use through different modes.

Potency of cannabis products, typically quantified as percentage of THC per weight of product, has also increased in recent decades. Several studies analyzing THC content in marijuana dry flower in the United States reported an increased potency of >10% in the last decade [6,[12], [13], [14]]. This increase in THC content may be a response to the rising demand for high-dose THC products [14,15]. For example, the sale of high-dose THC (>20% THC per weight) flower strains grew 50% between 2014 and 2016 in Washington State. An increase was also observed for the sale of cannabis extracts, for which average THC potency is triple that of products made from the cannabis flower [6] (Table 1).

## Cannabis Use by Reproductive-Aged Women

Although cannabis use is more prevalent among men than women, this gender gap is narrowing; 42% of people who use cannabis in North America are women, which is the highest proportion worldwide [1]. This relatively high-usage rate among North American women includes their reproductive years and during pregnancy and lactation [16,17]. The National Survey on Drug Use and Health (NSDUH) from 2002 through 2014 included surveys from of 200,510 women, 18–44 y of age. The population was described as 61% White, 17.2% Hispanic, 13.7% Black, and 8% other race/ethnicity; 59% had some college education; and 55.9% had annual family incomes <$50,000. Among the 10,587 pregnant women included in this survey, prevalence of past-month use of cannabis increased 62.4% between 2002 and 2014, although the frequency of use within this population was low (<4%) [17]. Data collected in the NSDUH also indicated that use rates during pregnancy increased substantially from 3.4% to 7.0% between 2002 and 2017, with the highest prevalence of daily marijuana use reported during the first trimester [18]. The 2017 annual Pregnancy Risk Assessment Monitoring System recorded information about the prevalence of cannabis use during the postpartum period in 7 states (Alaska, Illinois, Maine, New Mexico, New York, Pennsylvania, and West Virginia). They reported that overall prevalence of marijuana use among 4604 postpartum participants was 5.5%, with 4.1% of breastfeeding women reporting current use [19].

In contrast to overall usage trends, which are on the upswing, the perception of the dangers of cannabis use among women of reproductive age has declined [16,[20], [21], [22]]. In fact, cannabis products are occasionally viewed as representing a less harmful, more effective substance compared with over-the-counter and prescription medications [23,24]. Indeed, cannabis and cannabis-based products (containing THC) are often used [[24], [25], [26]]—and some dispensaries are advising them—for treatment of pregnancy-related morning sickness [27]. During breastfeeding, the main reasons reported by women for using cannabis include the treatment of health problems such as anxiety, depression, gastrointestinal problems (e.g., loss of appetite and nausea), chronic pain, and posttraumatic stress disorder [28,29].

Although very little is known about the potential effect of maternal cannabis use during pregnancy and lactation on the infant, THC has been detected in meconium of infants of mothers who use cannabis [[30], [31], [32]] and in human milk [[33], [34], [35], [36], [37], [38]], clearly indicating that the infant can be exposed to this and other compounds found in cannabis or metabolites thereof. Multiple studies in the last decade have associated prenatal cannabis exposure to negative newborn outcomes, such as low birthweight, preterm birth, or higher neonatal intensive care unit admission rates [32,[39], [40], [41], [42], [43]]. Longitudinal cohort studies have also associated prenatal cannabis exposure with poorer performances in verbal activities, short-term memory, or attention in infants and young adults [[44], [45], [46]]. However, inconsistent results have been observed in academic achievement, and it has been suggested that maternal/infant socioeconomic status might be mediating some of the relationships reported with poorer performance [47,48]. Nonetheless, research will need to be conducted to rigorously evaluate different forms of cannabis use (smoking, vaping, or ingesting) and to control for the amount of cannabis used to elucidate if there is a dose–response effect of prenatal cannabis exposure on infant outcomes.

There are even fewer studies evaluating the potential effects of maternal cannabis use on the infant during breastfeeding, and these studies have substantial limitations in that they include low numbers of participants and the co-use of alcohol, tobacco, and other drugs [49]. The scientific literature regarding the potential risks and benefits of cannabis use during breastfeeding is extremely limited and does not provide clear guidance [50]. Due to the limited evidence of effects of perinatal cannabis use, it is unclear whether the benefits of breastfeeding outweigh the potential risks of exposure to THC or its metabolites via breast milk. Organizations, such as the American Academy of Pediatrics, American College of Obstetricians and Gynecologists, the Academy of Breastfeeding Medicine, and the United States FDA recommend reduction or cessation of cannabis use by pregnant and breastfeeding women [49,[51], [52], [53]]. Nonetheless, it is clear with the increasing cannabis use trends that these organizations’ recommendations are not being followed. Furthermore, these recommendations are generally based on lack of evidence and extreme caution (precautionary principle) rather than scientific evidence of harm.

## Pharmacokinetics of Cannabinoids and Their Incorporation into Human Milk

Cannabinoids are grouped into 1 of 3 categories depending on their origin. They are referred to as phytocannabinoids if derived from the plant *Cannabis sativa*, endocannabinoids if produced in the human body, and synthetic cannabinoids if they are manufactured as “designer drugs” that bind to the cannabinoid receptors. To date, 125 cannabinoids have been isolated from the *Cannabis sativa* plant [54], among which THC and cannabidiol (CBD) are the most abundant and well-studied. THC is the compound in cannabis that produces many of its psychoactive effects, whereas CBD is a nonpsychoactive cannabinoid reported to have medicinal (e.g., analgesic, antispasmodic, and anti-inflammatory) properties [55,56].

The timing of cannabinoid appearance in the circulatory system after cannabis use varies widely and depends on the route of administration. Peak plasma THC concentration is achieved faster (within 3–10 min), and maximum concentrations in plasma are higher when the cannabis is inhaled compared with when it is ingested [56,57]. The amount of cannabinoids that can reach the circulatory system after inhalation ranges from 10% to 50% of the total present in the product used, although there is high interindividual variability related to depth of inhalation, puff duration, and length of breath hold [58,59]. When cannabis products are ingested, the appearance of THC in blood is slower, typically resulting in maximal circulating concentrations after 1 h [9].

**FIGURE 1:**
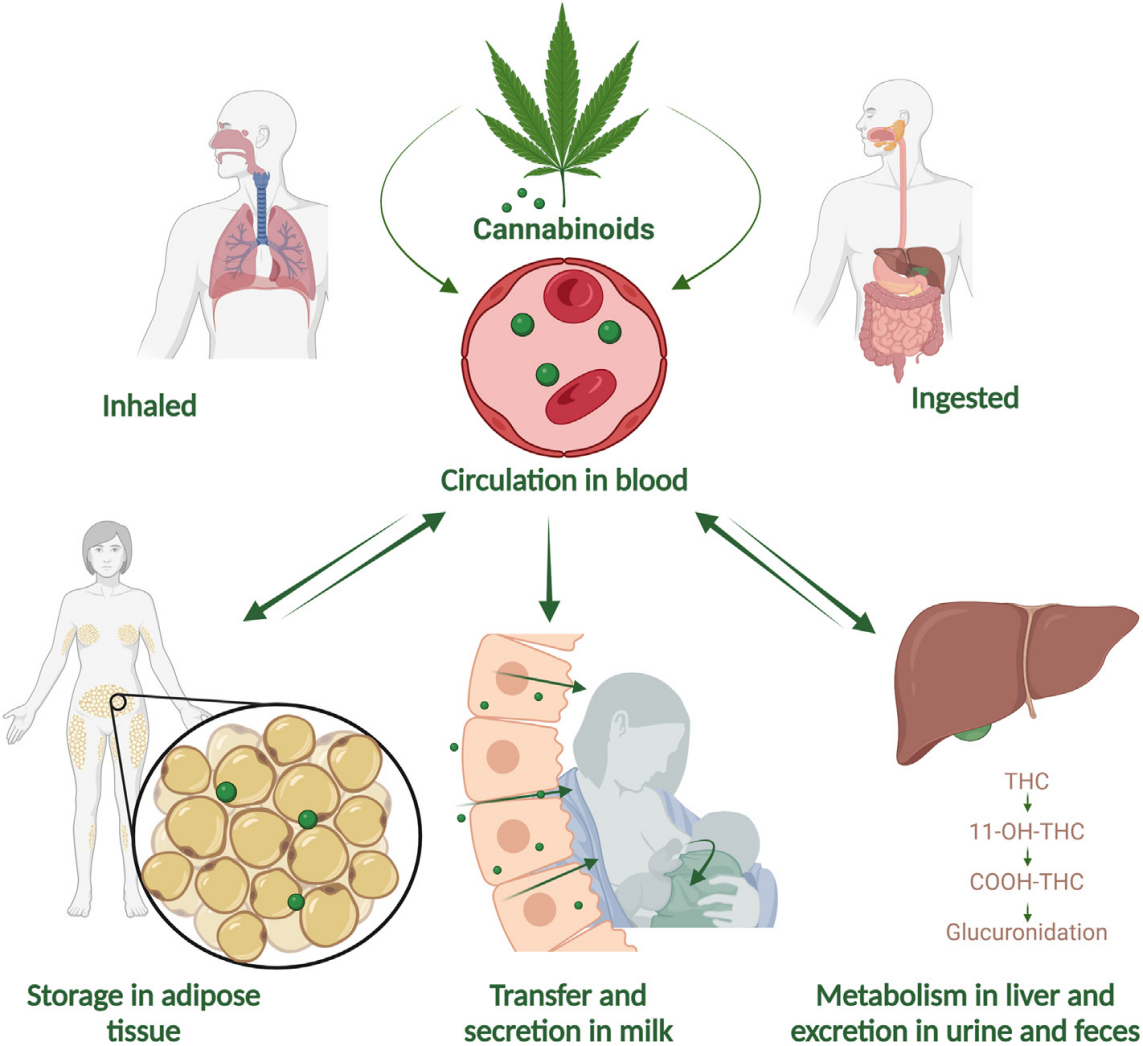
Absorption, transport, and metabolism of cannabinoids, including the most common routes of administration (inhaled and ingested) in relation to their presence in milk. Abbreviations: 11-OH-THC, 11-hydroxy-tetrahydrocannabinol; THC-COOH, carboxy-tetrahydrocannabinol; THC, Δ-9-tetrahydrocannabinol. Created with BioRender.com.

Approximately 90% of the THC in blood is partitioned into the plasma compartment, where it is almost entirely found bound to low-density lipoproteins [58]. Once in the blood, cannabinoids rapidly redistribute into well-vascularized organs and, due to their lipophilic nature, can easily be distributed to adipose tissue (Figure 1). Indeed, adipose tissue may be the major long-term accumulation depot, where fatty acid conjugates of THC and 11-hydroxy-tetrahydrocannabinol (11-OH-THC) may be formed, increasing their stability and reaching adipose-to-plasma ratios of up to 104:1 [58,59]. Therefore, it is highly likely that the amount and distribution of stored cannabinoids is affected by body composition [56], but results from the few studies that have investigated this have not been conclusive, as described below.

Ewell et al*.* [60] explored the potential association between body composition and pharmacokinetics of circulating THC after consumption of different edible marijuana products and found that none of the body composition characteristics (including age, height, bone mineral content, BMI, fat percentage and fat, and lean and total mass) were consistently related to pharmacokinetic parameters for any product. Wong et al. [61] assessed the potential effect of exercise on plasma concentrations of THC in people who regularly use cannabis and detected a positive correlation between BMI (generally used as an indirect indicator of adiposity) and exercise-induced increased concentration of plasma THC. Whereas higher BMI might represent greater adipose tissue mass (which could therefore store greater amounts of lipophilic cannabinoids), high BMI can also reflect greater muscle mass. Therefore, more studies are needed to describe how body composition, as assessed by more sophisticated methods such as dual x-ray absorptiometry, might influence cannabinoid pharmacokinetics and whether these relationships are similar across the lifespan. Indeed, pregnancy and lactation are stages of particular interest to this concern. The significant changes in body fat mass that occur during pregnancy and lactation may have different effects on THC circulation than static body fat mass.

In addition to their high lipid-solubility, cannabinoids have a small molecular weight (314.46 g/mol), allowing them to transfer into and become concentrated within lipid-rich tissues, such as the breast [34] (Figure 1). Indeed, several studies [[33], [34], [35], [36], [37], [38],^62^,^63^] have demonstrated the presence of phytocannabinoids and their metabolites in human milk (Table 2). Relative incorporation of cannabinoids into milk appears to be different depending on time postpartum. During the first days of lactation, milk-producing mammary epithelial cells (MECs, sometimes referred to as lactocytes) are small, and the spaces between them are wide. These wide intercellular gaps allow leukocytes, Ig, proteins, and drugs (including cannabinoids) to transfer easily from the mother’s circulation into the milk [64]. Once the intercellular gaps (sometimes referred to as leaky tight junctions) are reduced in size, the transfer of drugs and other large molecules into the milk occurs by passive or facilitated diffusion down their concentration gradients [65].

**TABLE 2:** Published research reporting concentrations of cannabinoids and their metabolites in milk produced by women who use cannabis.

Reference	*n*	Timing and frequency of cannabis use	Milk collection methodology						Cannabinoids and metabolites concentrations in milk, median (IQR) or specified units
			Time PP	Excl. BF	Collection method	Collection time	Full exp.	Single/both breast	
Perez-Reyes and Wall, 1982 [36]	2	Subject 1: 1/d Subject 2: 7/d	7–8 mo	NR	NR	NR	NR	NR	Subject 1: 105 ng/mL THC; 11-OH-THC and 9-carboxy-THC were not detected. Subject 2: 340 and 4 ng/mL THC and 11-OH-THC, respectively; 9-carboxy-THC not detected.
Marchei et al., 2011 [62]	1	NR	NR	NR	NR	NR	NR	NR	86 and 5 ng/mL THC and 11-OH-THC, respectively.
Baker et al., 2018 [33]	8	0.025–1 g/d Abstained for 24 h before using 0.1 g of dry flower	3–5 mo	Yes	Pump	20 min and 1, 2, and 4 h after use	-	Both	Mean and maximum (1 h after use) THC: 53.5 and 94 ng/mL, respectively.
Bertrand et al., 2018 [34]	50	88% of subjects used at least daily	2/3 of women <1 y	NR	NR	NR	Yes	NR	THC: 9.47 (IQR: 2.29–46.78) ng/mL (*n* = 34) 11-OH-THC: 2.38 (IQR: 1.35–5.45) ng/mL (*n* = 5) CBD: 4.99 (IQR: 2.92–5.97) ng/mL (*n* = 5)
Moss et al., 2021 [35]	20	Used daily. Last use <48 h before sample collection	<2 mo	NR	NR	2 wk and 2 mo PP	No	NR	THC: 27.5 (IQR: 0.9–190.4) ng/mL (*n* = 34) 11-OH-THC: 1.4 (IQR: 0.7–5.2) ng/mL (*n* = 5) THC-COOH: 1.9 (IQR: 0.5–16.6) ng/mL (*n* = 18) CBD: 1.2 (IQR: 0.5–17.0) ng/mL (*n* = 13)
Sempio et al., 2021 [37]	30	Used during pregnancy; frequency not provided	NR	NR	NR	NR	NR	NR	THC: 8.08 (range: 0.84–130) ng/mL (*n* = 30) 11-OH-THC: 1.94 (range: 1.66–2.42) ng/mL (*n* = 5) THC-COOH: 1.30 (range: 2.05–2.98) ng/mL (*n* = 7)
Wymore et al., 2021 [38]	25	Prenatal use >2 times/wk	1–6 wk	NR	NR	Through first 6 wk PP	NR	NR	THC in all samples: 3.2 (IQR: 1.2, 6.8), 5.5 (IQR: 4.4, 16.0), and 1.9 (IQR: 1.1, 4.3) ng/mL in 1, 2, and 6 wk, respectively
Josan et al., 2022 [63]	13	Daily use during pregnancy (*n* = 12) and PP period (*n* = 8)	6–8 wk	NR	Pump	From daily or weekly pumped	NR	NR	THC: 22 (range: 0.625–503) ng/mL (*n* = 13) 11-OH-THC: 6 (range: 4–22) ng/mL (*n* = 3) THC-COOH: 2.6 (range: 1.6–5.9) ng/mL (*n* = 5)

Abbreviations: 11-OH-THC, 11-hydroxy-tetrahydrocannabinol; BF, breastfeeding; CBD, cannabidiol; excl., exclusively; exp., expression; IQR, interquartile range; NR, not reported; PP, postpartum; THC, Δ-9-tetrahydrocannabinol; THC-COOH, carboxy-tetrahydrocannabinol.

The passage of drugs into milk is affected by many additional factors, such as acid–base dissociation constant (pKa) and protein binding [64]. Due to its lipophilic nature and small molecular weight, THC transfers particularly readily into milk. A pharmacokinetic study carried out by Baker et al. [33] demonstrated that, after maternal cannabis inhalation, 2.5% (range: 0.4%–8.7%) of the THC dose was secreted in the milk. Once in milk, THC can be partially ionized because of its basic pKa (10.6) and is therefore not able to pass back into maternal plasma through passive diffusion even when its concentration is greater in milk than in maternal plasma [65]. This phenomenon leads to accumulation of THC in the milk, explaining the high milk-to-plasma ratios (up to 8:1) reported previously [36,38]. Moreover, cannabis metabolites stored in adipose tissue may have a slow release into the blood circulation and eventually milk during lactation because these compounds can be detected in milk from women who frequently use cannabis (>2 times/wk) even after several weeks of abstention [38].

THC circulates through the body via the bloodstream, eventually reaching the liver. Metabolism of THC includes hydroxylation in the liver through cytochrome P450, resulting in 11-OH-THC, which also has psychoactive effects. Plasma concentrations of 11-OH-THC after oral ingestion are greater than those observed in the plasma after smoking cannabis [58]. Degradation of THC by gastric secretions (e.g., hydrochloric acid), combined with first-pass liver metabolism, reduces bioavailability of THC to 6%–7% of the ingested dose [58,59]. Further oxidation of 11-OH-THC produces carboxy-tetrahydrocannabinol (THC-COOH), which is eventually glucuronidated [58]. Most of the cannabinoids and their metabolites are excreted in feces (65%–80%), with the remainder in urine (20%–35%) [57]. The metabolites excreted in feces—mainly in the form of nonconjugated metabolites—can be detected for over a week after use [[66], [67], [68], [69]]. Conversely, metabolites in urine are mainly excreted in conjugated form, and a single dose of THC may result in measurable metabolites in urine for ≤12 d. Nevertheless, substantial differences can be observed in the period of excretion depending on use frequency; although cannabis cannabinoid metabolites can be detected in urine for (on average) 8.5 d after use in people who occasionally use cannabis, average time for excretion is 19 d in people who frequently use cannabis [58].

## Cannabinoid Receptors in Mammary Epithelial Cells

The physiologic and psychoactive effects of cannabis use are a result of the interaction of phytocannabinoids with the endocannabinoid system (ECS). This system is composed of cannabinoid receptors (detailed below), their endogenous ligands (mainly anandamide [N-arachidonoylethanolamide [AEA]) and 2-arachidonoylglycerol) and the enzymes responsible for endocannabinoid synthesis, reuptake, and degradation (fatty acid amide hydrolase and mono-acyl glycerol lipase) [70].

The main receptors in this system are G protein-coupled receptors (GPRs) referred to as cannabinoid type 1 receptors (CB1Rs) and cannabinoid type 2 receptors (CB2Rs), although cannabinoids can also bind to GPR55, GPR18, GPR3, GPR6, and GPR12. CB1R is particularly abundant in certain regions of the central nervous system, such as the frontal cortex, hippocampus, basal ganglia, and cerebellum. Decreased presence of CB1R in the central nervous system is associated with disruption of its many roles such as control of motor function, cognition and memory, and analgesia [71]. Some studies also provide evidence for presence of CB1R in peripheral tissues, such as adrenal glands, pituitary gland, heart, lung, prostate, liver, uterus, ovary, testis, bone marrow, thymus, and tonsils [[72], [73], [74]], as well as adipose tissue, pancreas, and skeletal muscle [75]. Conversely, CB2Rs are abundantly present in peripheral organs and cells with immune function, including macrophages, the spleen, tonsils, thymus, and leukocytes, as well as the lungs and testes [71].

The presence of CB1R and CB2R in MECs has been described mostly in studies related to breast cancer, as CB2R is overexpressed in some subtypes of mammary gland tumors [76]. Only a limited number of studies, however, have reported the presence (or absence) of cannabinoid receptors in MECs of healthy women. Josan et al. [77] detected a higher expression of the cannabinoid receptor 2 gene (*CNR2*) in differentiated HC11 cells (a mouse MEC line) than in undifferentiated cells. Regarding human cell lines, Caffarel et al. [78] evaluated expression of CB1R and CB2R in healthy human MECs as well as in several human breast cancer cell lines by real-time quantitative PCR and confocal microscopy. Higher concentrations of CB1R mRNA were detected in healthy MECs compared with cancerous breast tissue, whereas the opposite was observed for CB2R mRNA. Qamri et al. [79] also investigated the presence of CB1R and CB2R in healthy human MECs and several breast cancer cell lines using tissue microarray. Expression of both receptors was detected in all cancer cell lines assessed as well as in healthy MEC samples. However, they reported a higher percentage of CB1R and CB2R staining in breast cancer cell lines (28% and 35%, respectively) than in healthy MECs (3% and 5%, respectively).

The expression of the cannabinoid receptor genes *CNR1* and *CNR2* in *lactating* human MECs has been proved in a limited number of studies [80,81]. Lemay et al. [81] sequenced mRNA found in the milk fat layer from 3 colostrum, 5 transitional, and 8 mature milk samples. *CNR1* and *CNR2* genes were expressed in 1 colostrum sample and 1 mature milk sample, respectively. Twigger et al. [80] analyzed mammary cells isolated from human milk (*n* = 8) and nonlactating mammary tissue (*n* = 7) using single-cell transcriptomic methodology. Although the expression of *CNR1* was low overall, higher expression was observed in nonlactating mammary tissue samples than in human milk samples. Expression of *CNR2* was low and similar in both groups of samples [80]. Further research with a larger number of samples is necessary to disclose if and how the expression of *CNR1* and *CNR2* might be affected by the lactation process.

Cannabinoids can also bind to peroxisome proliferator-activated receptors (PPARs) [57,82], a family of nuclear hormone receptors (including isoforms α, β/δ, and γ) that can bind to DNA sequences, leading to changes in the transcription of target genes. Although PPARs are primarily activated by unsaturated fatty acids [83], it has been suggested that cannabinoids can also activate PPAR in different ways. For example, cannabinoids can *1*) be transported intracellularly by fatty acid-binding proteins (FABP) and activate PPAR directly; *2*) activate surface cannabinoid receptors causing intracellular signaling cascades that lead to the activation of PPAR indirectly; and *3*) be metabolized into cannabinoid metabolites, which can activate PPAR [[84], [85], [86]].

PPARs have been detected mainly in adipose tissue and, to a lesser extent, in mammary tissue of several species. Studies in cattle have demonstrated that the gene peroxisome proliferator-activated receptor γ (*PPARG*) is highly expressed in adipose tissue and moderately expressed in mammary tissue and in a mammary epithelial cell line (MAC-T) [87,88]. Moreover, expression of *PPARG* in the mammary gland increases during pregnancy and lactation [88,89]. The *PPARG* gene has been found to be expressed also (in low-to-moderate amounts) in lungs, spleen, ovaries and, at very low concentrations, in liver, kidneys, leukocytes, and small intestine [87,90,91]. In monogastrics such as mice and humans, *PPARG* is also highly expressed in adipose tissue [[92], [93], [94], [95]].

The expression of the gene peroxisome proliferator-activated receptor α (*PPARA*) is more widespread among tissues and cells than that of *PPARG*. The highest expression of *PPARA* has been described in the kidney and liver, followed by adipose tissue, small intestine, and semitendinosus muscle in dairy cattle. Mammary glands of cattle have a relatively modest expression of *PPARA* [90]. PPARβ/δ is the least studied PPAR isotype. Bionaz et al. [90] observed similar expression of the gene peroxisome proliferator-activated receptor δ (*PPARD)* in all bovine tissues and cells assessed (including adipose tissue, rumen, jejunum, liver, kidney, muscle, hoof, lung, mammary gland, and placenta). Expression of *PPARD* in bovine mammary cells has also been demonstrated by others [96].

As mentioned above, cannabinoids can be transported intracellularly bound to FABPs, proteins that mainly mediate the cytosolic movement of lipids [86]. FABPs are found in numerous cell types of mammalian tissues involved in the uptake and/or utilization of fatty acids [97]. Indeed, high concentrations of FABPs have been found in rat [98,99] and bovine [100] mammary tissue.

In addition, transient receptor potential channels are highly selective epithelial calcium (Ca^2+^) channels involved in ionic homeostasis and cellular signaling [101]. Cannabinoids can bind to members of the transient receptor potential vanilloid channel subfamily expressed in healthy human breast tissues [102]. Cannabinoids can also bind to monoamine transporters, adenosine equilibrative nucleoside transporters, and glycine receptors [57,82] whose possible effects on lactogenesis and milk synthesis are currently unknown.

As previously discussed, there are several pathways through which cannabinoids might interfere with MEC function. However, the mammary gland is a complex matrix made up of MECs, stroma cells, adipose tissue, blood and lymphatic vessels, and nerve tissue [103]. Consequently, studying the effects of cannabinoids in isolated MECs does not fully replicate the environment of the human mammary gland. Indeed, use of a more naturalistic and complex tissue matrix that includes adipose cells would be more translatable. The use of organoids, for example, is an important model for human mammary gland research [104] because 3-dimensional mammary organoid models reproduce extracellular conditions, maintain spatial morphology, and could be cocultured with adipocytes and other stromal components [104]. Moreover, organoids can be maintained in controlled medium, thus inducing the lactating conditions and in turn making it possible to understand the effects of cannabinoids on the full scope of the lactating mammary gland [105,106].

## Effects of Cannabinoids on Milk Composition

**FIGURE 2:**
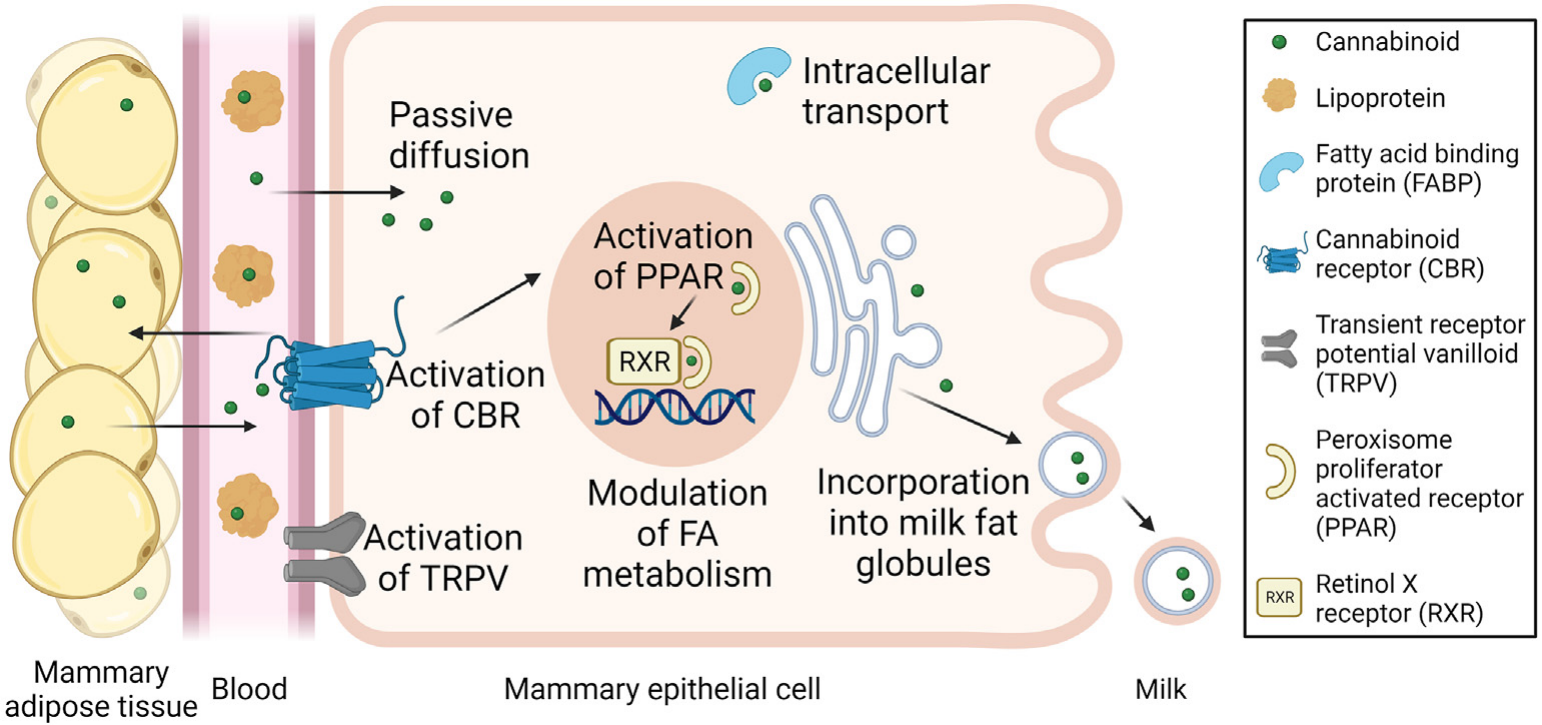
Putative effects of cannabinoids in mammary epithelial cells and secretion of cannabinoids in milk. Abbreviations: CBR, cannabinoid receptor; FA, fatty acid; FABP, fatty acid-binding protein; PPAR, peroxisome proliferator-activated receptor; RXR, retinol X receptor; TRPV, transient receptor potential vanilloid. Created with BioRender.com.

Roles of the ECS include regulating several physiologic processes such as homeostasis and energy balance, as well as modulation of numerous neuro-processes, including anxiety, feeding behavior/appetite, emotional behavior, depression, neurogenesis, neuroprotection, reward, cognition, learning, memory, and pain sensation [55,107]. Regarding the possible relationship of the ECS with milk composition, one of the most closely linked potential roles of the ECS is its likely modulation of lipid metabolism. This is because modulation of lipid metabolism is carried out in part through direct activation of PPARs or through the activation of cannabinoid receptors (Figure 2), which in turn triggers a cascade of reactions that ultimately activate PPARs (108). PPARs activated by cannabinoid form a heterodimer with the retinoid X receptor (RXR). This PPAR/RXR complex can bind to specific response elements in the DNA, in turn regulating expression of target genes involved in lipid metabolism and cell differentiation [109]. PPARγ is upregulated in mammary tissue during lactation, suggesting an essential role in regulation of milk fat synthesis [110].

In addition, the endocannabinoid AEA acts as an agonist of both CB1R and PPARγ, causing adipocyte differentiation and lipid accumulation [111,112]. Treatment of mouse 3T3-F442A preadipocytes with the CB1R receptor agonist HU210—a synthetic cannabinoid—increased the concentration of PPARγ and the accumulation of lipid droplets [113]. The same effect was observed after treating mouse adipocytes with AEA; increased *PPARG2* gene expression and CB1R protein content were observed compared with untreated cells [112]. Moreover, activation of adipocyte CB1Rs in mice stimulates lipoprotein lipase activity [114]. This enzyme plays an important role in milk fat synthesis as it regulates the hydrolysis of circulating triglyceride and the uptake of fatty acids in the mammary gland [115]. Accordingly, blocking CB1R binding in adipose tissue increases lipolysis, decreases lipogenesis, and increases the oxidation of fatty acids inside the adipocyte [108].

In addition to endocannabinoids, some polyunsaturated fatty acids such as some isomers of conjugated linoleic acid (CLA) can act as natural PPARγ ligands. Indeed, the effect of *t*10*c*12-CLA on lipid milk composition—both in animals and humans—has been described previously. More specifically, treatment with PPARγ ligand *t*10*c*12-CLA has a clear negative effect on lipogenic networks in several species such as cattle [91,116], mice [117], goats [118], and humans [119].

Whether cannabinoids have the same effect is currently unknown, but as described above, cannabinoids might modulate milk macronutrient composition, especially lipid and fatty acid content, by activating cannabinoid receptors and/or PPARγ. However, only a limited number of studies have addressed the direct effect of phytocannabinoids on milk composition, and additional rigorous research is warranted.

In a recent in vitro study, the effect of THC and CBD on milk production was investigated using HC11 cells (a MEC line isolated from mice). After treating HC11 cells with different amounts of either THC or CBD, reduced transcription (lower mRNA concentrations) of the protein synthetic genes casein β (*CSN2*) and whey acidic protein was observed for both cannabinoids. THC and CBD treatments also decreased expression of several genes related to lipid metabolism (fatty acid synthase [*FASN*], fatty acid-binding protein 4 [*FABP4*], glucose transporter 1 [*GLUT1*], hexokinase 2 [*HK2*], perilipin 2 [*PLIN2*], and lipoprotein lipase [*LPL*]) and reduced overall lipid concentrations [77]. These results suggest that cannabinoids may affect human milk macronutrient composition. However, the effect of cannabis or its metabolites on the lactating mammary gland needs to be confirmed with in vivo and clinical studies.

In humans, only one study has investigated the association between cannabis use and milk composition. In the study conducted by Josan et al. [63], a single milk sample was collected from 19 breastfeeding women who used cannabis and 17 breastfeeding women who did not use cannabis over 6–8 wk postpartum. The participants provided 2 ounces of milk from their daily or weekly expression, using either manual or electric pumps. Milk samples were analyzed for several cannabinoids by HPLC-mass spectrometry; lactose using an ELISA kit; and lipids, total carbohydrates (as sum of lactose and oligosaccharides), and crude protein (including nonprotein nitrogen and true protein) from nitrogen content using a Miris Human Milk Analyzer. No differences were observed in concentrations of lipids, total carbohydrates, crude protein, or true protein in milk produced by women who used compared with those who did not use cannabis. However, lactose concentrations were higher in the milk produced by women who used only cannabis (not mixed with tobacco) compared with those of women who did not use. When controlling for cooccurrence of tobacco use, milk produced by women who used cannabis and tobacco had lower concentrations of total carbohydrates and higher concentrations of crude and true protein [63]. Because sampling details were not provided (unknown amount of foremilk and hindmilk) and timing of sampling was not controlled for a variety of important factors (e.g., time of day and time since last feed) related to variation in milk composition (e.g., lipid content), these data should be interpreted with caution. In addition, time since last cannabis exposure was not recorded. Clearly, additional well-controlled studies are needed to understand the potential effects of maternal cannabis use on milk composition.

## Maternal Cannabis Use and Milk Production

**FIGURE 3:**
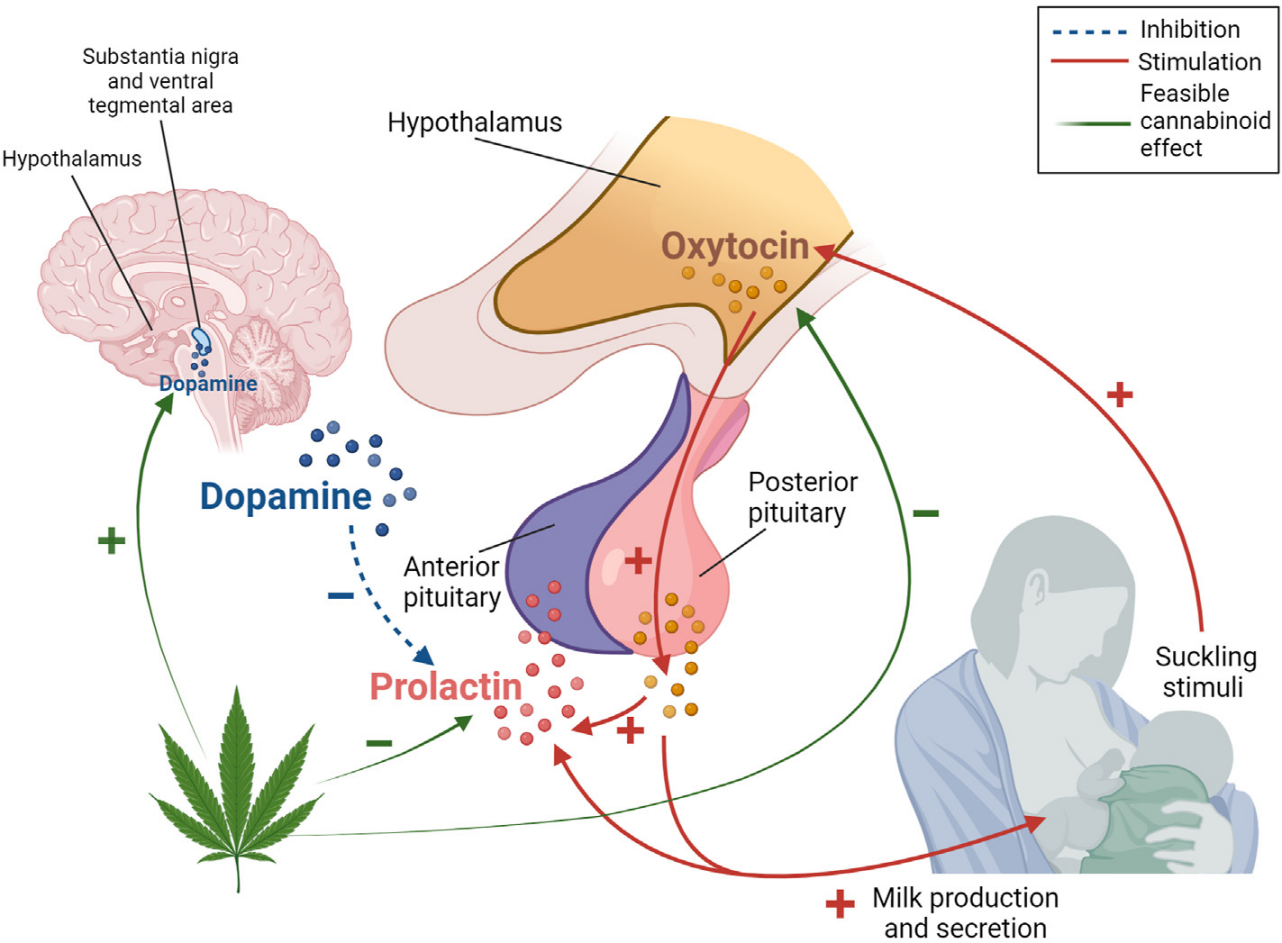
Feasible effects of cannabinoids on the hypothalamus–pituitary axis related to lactation. Created with BioRender.com.

The endocrine system is responsible for regulating lactation, including mammogenesis (development of the mammary gland), lactogenesis (establishment of lactation), and galactopoiesis (maintenance of milk secretion). Milk production is controlled by prolactin (PRL) and growth hormone (GH), although regulation of milk production is slightly different depending on the species. GH is dominant in ruminants, whereas PRL is dominant in rodents and humans [120,121]. In humans, lactogenesis starts in midpregnancy with the differentiation of the mammary gland to secrete small quantities of specific milk components through a combination of high concentrations of estrogen and progesterone. However, secretion of milk is inhibited by progesterone at this point. The second step in lactogenesis occurs around parturition; after a rapid decrease of progesterone concentrations, inhibition of PRL release ends and milk production and secretion start. The suckling action of the infant at the breast (or pumping) causes release of oxytocin in the hypothalamus that stimulates milk ejection in the mammary gland and the release of PRL by the anterior pituitary gland (Figure 3). Conversely, secretion of dopamine and/or the lack of suckling stimuli (or pumping) inhibit PRL release, and consequently, milk production [120,122].

Cannabinoids such as THC and CBD are known to interact with the dopaminergic system [123,124], leading to possible effects on milk synthesis. However, studies to determine how cannabinoids interact with the dopaminergic system [[125], [126], [127], [128]] have yielded inconclusive results (described below), and more research is warranted.

### Effects of cannabinoids on PRL

The inhibitory effect of endocannabinoids AEA and 2-arachidonoylglycerol on PRL has been demonstrated in mice [74,129,130], and a similar effect was observed after THC administration [131,132]. Rodríguez de Fonseca et al. [125] observed a brief rise in plasma PRL before a prolonged decrease after THC administration in rats. Pagotto et al. [126] proposed a biphasic action of cannabinoids on PRL secretion; first an increase of PRL secretion by activation of CB1R in the pituitary, followed by an inhibitory effect through the activation of dopamine release. However, studies in monkeys have shown both a reduction of circulating PRL concentrations [127] and no clear trends [128] after THC administration.

The specific effect of THC on PRL was addressed in 2 studies on rats carried out in the 1980s. In the first study, THC was injected to ovariectomized rats, and serum PRL concentrations were assessed before and after treatment. The research reported that THC suppressed serum PRL concentrations 10 min after the treatment, an effect that lasted for 1 h [133].

In the second study, THC or vehicle was injected into lactating rats before initiating suckling or once suckling was established [134]. After treatment with vehicle, serum PRL concentrations increased from 14 to 215 ng/mL during suckling and decreased to 74 ng/mL 1 h after suckling ceased. Conversely, in the THC group, PRL concentration did not change during suckling, reaching the highest concentration at 53 ng/mL. When testing the THC effect once suckling was established, they observed that serum PRL concentration declined following treatment, and concentrations remained lower even though the suckling continued. Whether the THC effects on serum PRL concentrations observed in lactating rats also occur in lactating women has not been explored.

The effect of THC on human serum PRL concentrations in nonlactating women using cannabis has been studied, but results are conflicting. Some studies have concluded that THC does not affect serum PRL concentration after acute exposition [[135], [136], [137]]. However, D’Souza et al. [136] observed that plasma PRL in a group of people who frequently used cannabis (>10 exposures over last month) was lower than in people who did not use. On the contrary, Lee et al. [138] quantified PRL in the serum of 6 people who chronically and heavily used cannabis (used for ≥5 y, and between 25 and 30 d of use within the last months) and reported that half had elevated PRL concentrations beyond the reference range.

The association of cannabis use and milk production was considered in one study carried out by Josan et al. [63]. Lactating women over 6–8 wk postpartum who used cannabis (*n* = 22) and others who did not use cannabis (*n* = 18) completed a survey about breastfeeding practices and usage patterns of cannabis. The 66.7% of the women who used cannabis during pregnancy used it daily, mostly via smoking (94%). Postpartum use was reported by 55%, following similar patterns; 66.7% used cannabis daily, and most (83%) smoked [63]. In this study, the group of women who used cannabis self-reported lower concentrations of milk production (average amount of milk pumped at one time) during the first 6 wk postpartum. It is important to note that, 41% and 36.4% of the participants who used cannabis reported cigarette use during and after pregnancy, respectively, and reduced production of milk among tobacco-smoking women has been reported [139]. Therefore, it is still unknown whether cannabis use itself does indeed influence milk production.

The possible reduction in milk production caused by cannabinoids’ effects on PRL concentrations could be a factor affecting the establishment of breastfeeding. Indeed, concerns about milk supply is one of the main reasons why women stop breastfeeding completely before 6 mo [140,141]. Although a shorter duration of breastfeeding was observed in women who used cannabis than in those who did not use [142], the association of this shorter breastfeeding duration with lower milk production was not addressed in the study.

To evaluate the possible effect of cannabinoids on milk production, accurate methods to measure milk production must be used to achieve reliable results. Factors influencing variability in milk production, such as time postpartum, time of the day, time since last breastfeeding session, and maternal factors (e.g., adiposity and medications) must be controlled for or at least detailed. Likewise, an accurate analytical method for measuring milk production, such as deuterium dilution or test-weighing, needs to be used to fully understand the physiology and outcomes that might be associated with cannabis use during breastfeeding [143]. Thus, studies designed to determine the specific effect of cannabis (and not other products such as tobacco) on milk production should be pursued.

### Effects of cannabinoids on dopamine and oxytocin

Increased dopamine release caused by cannabinoids has been observed in studies using murine models [144,145]. Moreover, it has been suggested that this cannabis-induced release of dopamine is caused by the activation of CB1R in the hypothalamus [146,147]. Higher dopamine concentrations might inhibit PRL release from the pituitary, affecting regulation of milk synthesis and secretion [148].

The effect of cannabinoids on oxytocin during lactation was assessed in an animal model study. Intravenous THC or vehicle was injected into lactating rats and the stretch response of the pups—a signal of milk ejection—was recorded [149]. Milk ejection is caused by an increase in the mammary pressure provoked by the release of oxytocin. The intervals between milk ejections before THC or vehicle injection were 7 and 6.5 min, respectively. After injection of vehicle in the control group, intervals between milk ejections remained similar to those before treatment. However, after injection of THC, there was a latency period of 59 min before the next milk ejection was observed; even after resuming milk ejections, intervals were lengthened (between 15 and 16 min).

In addition to regulating lactogenesis, oxytocin and dopamine play key roles in establishment of the mother–infant bond. In animal models, higher oxytocin receptor concentrations have been observed in rats that showed more attachment to the pups. The same effect has been observed with dopamine; decreased dopamine transporters are associated with weakened maternal behavior [150]. A recent study demonstrated that the ECS is involved in the oxytocin-dependent formation of pair bonds. The administration of an endocannabinoid antagonist to female paired prairie voles inhibited the oxytocin-bonding effect and even increased “rejection-like” behaviors toward their partners [151]. The possible effect of cannabis use on mother–infant bonding has yet to be explored.

## Summary

Prevalence of cannabis use among the United States population has increased over the last 20 y with a notable proportion of pregnant and breastfeeding women reporting use. At the same time, perceived risk of cannabis has decreased within this population. It is widely demonstrated that phytocannabinoids can be found in milk produced by women who use cannabis during pregnancy and/or breastfeeding. Although empirical support in human is lacking, results from animal studies and human studies focused on nonpregnant, nonlactating individuals suggests the possibility that long-term accumulation of cannabinoids in mammary adipose tissue might interfere with the process of lactogenesis by directly activating cannabinoid receptors. Furthermore, phytocannabinoids may affect milk output and composition, especially lipid and fatty acid profiles, by modulating the expression of genes involved in fatty acid synthesis. Regulation of synthesis and secretion of milk might also be affected by cannabinoids via their interactions with the endocrine system. However, substantial research is needed utilizing rigorous methods for milk collection and production to demonstrate any of these potential effects.

## Acknowledgments

The authors would like to thank Isabel Marlens from the University of Idaho Writing Center for her support in the drafting of this manuscript.

## Author contributions

The authors’ responsibilities were as follows – ICN, MKM, MAM: conceptualized and designed this review; ICN: researched, analyzed the background literature, and drafted the manuscript; MKM, MAM, JEW, EAH, CLM: provided critical review, commentary, and revisions to the manuscript; and all authors: read and approved the final manuscript.

## Conflict of interest

The authors report no conflict of interest.

## Funding

ICN is funded by The University of Idaho P3-R1 Grant Matching Program.

## Data availability

Data for this review will be made available upon written request to the corresponding author.
